# Popular Cyclopædia of Natural Science—Vegetable Physiology

**Published:** 1841-07

**Authors:** 


Art. VIII.—*Popular Cyclopædia of Natural Science—Vegetable Physiology.* Published by the Society for the Promotion of Popular Instruction.—*London*, 1841. 8vo, pp. 295.

We know nothing of, and indeed never heard of, the Society by which this small volume is said to be published; but from which we are taught to expect, from the prospectus, nine or ten more volumes of the same general character as the present, to appear quarterly. We can however say conscientiously, after an examination of the first part now before us, that if the subsequent volumes are only nearly as good as this, the Society will, by their labours, have conferred a great benefit on the rising generation. We know not when we have met with a work more clearly and pleasantly written, more full of good matter with so little rubbish of any kind, or one better adapted to fulfil the object for which it is intended. We agree in every word of the following statement prefixed to the work by the Society, and cordially recommend the treatise to the notice of all our readers, who on their own account, or that of their sons or daughters, may be interested in the study of vegetable physiology:

"The 'Society for the Promotion of Popular Instruction' have much pleasure in offering to the public the following treatise on vegetable physiology, as the first-published portion of their 'Popular Cyclopædia of Natural Science;' exhibiting, as it does, the successful carrying-out of the principles set forth in the Prospectus of the series; and, at the same time, completely harmonizing with the general objects of the society. They feel assured that it will be found sufficiently simple in its character, and clear in its explanations, to be regarded as an elementary treatise, adapted to those who have no previous knowledge of the subject; whilst its systematic arrangement, and the scientific value of the principles laid down in it, render it an excellent introduction to more comprehensive works on the same subject. The general reader, who seeks no more than entertainment or recreation, will find it in this volume, in the copious illustrative facts and interesting collateral information, with which it abounds: whilst to the agriculturist, the gardener, and the domestic economist, it supplies principles and practical applications of great importance."

